# Undergraduate nursing students' attitudes and needs regarding the use of generative artificial intelligence in professional learning: a qualitative study

**DOI:** 10.3389/fpubh.2026.1854814

**Published:** 2026-06-10

**Authors:** Mengquan Qiao, Hongqin Zhang, Yanmeng Shen, Tao Zhang, Shuai Liu, Yan Lou, Lingling Yang, Chunmei Song, Jianping Liu

**Affiliations:** 1 School of Nursing, Tianjin Medical University, Tianjin, China; 2 Second Clinical Medical College, Tianjin Medical University, Tianjin, China; 3 First Affiliated Hospital of Henan University of Chinese Medicine, Zhengzhou, China; 4 Department of Neurology, Yancheng Third People's Hospital, The Affiliated Hospital of Jiangsu Medical College, Yancheng, China; 5 Department of Neurology, Affiliated Hospital No. 6 of Nantong University, Nantong, China; 6 Department of Disinfection Supply Center, Yancheng Third People's Hospital, The Affiliated Hospital of Jiangsu Medical College, Yancheng, China; 7 Department of Disinfection Supply Center, Affiliated Hospital No. 6 of Nantong University, Nantong, China

**Keywords:** attitudes, generative AI, needs, nursing education, qualitative study, undergraduate nursing students

## Abstract

**Objective:**

This study aimed to explore undergraduate nursing students ‘attitudes toward and needs regarding the use of generative artificial intelligence (AI) in professional learning, to provide a reference for the better integration of generative AI into nursing education.

**Methods:**

This study employed a qualitative research design. Guided by Colaizzi's seven-step method, semi-structured in-depth interviews were conducted with 18 undergraduate nursing students with experience in using generative AI. Participants were recruited through purposive sampling from three universities in China between January and March 2026.

**Results:**

Two themes and six subthemes were identified: (1) undergraduate nursing students have a rational attitude toward generative AI, including broadening learning perspectives and improving learning efficiency, alleviating psychological anxiety, and existing concerns; (2) undergraduate nursing students ‘needs regarding the use of generative AI in professional learning, including information accuracy, humanistic care, and external support.

**Conclusion:**

Undergraduate nursing students generally adopt a rational and accepting attitude toward generative AI. While recognizing its value as a learning aid, they maintain a clear understanding of its limitations and have expressed a pressing need for accurate information, humanistic care, and external support. Nursing institutions should strengthen the regulation, guidance, and integration of generative AI into teaching to promote the high-quality training of nursing professionals.

## 1 Introduction

In recent years, with the rapid advancement of artificial intelligence technology, generative AI—represented by platforms such as ChatGPT and DeepSeek—has rapidly penetrated the field of higher education due to its strengths in logical reasoning and personalized interaction. This has disrupted traditional models of classroom instruction, self-directed learning, and practical training, driving profound transformations in higher education (1, 2). As a discipline that combines theory, practice, and humanistic elements, nursing offers tremendous potential for the application of generative artificial intelligence (3). In China, to meet the demand for high-quality nursing care, higher nursing education has developed rapidly; between 2002 and 2018, the number of nurses with a bachelor's degree increased twentyfold (4). The core objective of the program is to cultivate well-rounded nursing professionals equipped with solid professional knowledge, standardized clinical skills, strong humanistic qualities, and critical thinking abilities. The integration of generative AI can expand access to medical information and, to a certain extent, enhance nursing students' learning efficiency and clinical reasoning skills (2, 5). As a tool to support innovation, generative AI has greatly facilitated the academic studies of undergraduate nursing students. An increasing number of undergraduate nursing students are applying generative AI to their coursework (6, 7), to help them devise solutions to problems, receive personalized support (7); receive guidance on academic writing (8) and better understand complex medical concepts (9). As the backbone of the future nursing workforce, undergraduate nursing students' attitudes toward generative AI technology, their actual user experiences, and their professional needs all influence the quality of nursing education under current development models. Although generative AI holds great potential in nursing education—effectively addressing issues such as insufficient teacher-student interaction, limited practical settings, and uneven learning progress that plague traditional teaching models—its application also presents certain drawbacks. For instance, prolonged use of generative AI can lead to excessive reliance among nursing students, thereby diminishing their critical thinking skills (2, 10); it may generate inaccurate information (2); and it may also carry risks of ethical controversies and privacy breaches (6). Currently, research on generative AI in nursing education, both domestically and internationally, has largely focused on evaluating learning outcomes, exploring application pathways, and assessing academic performance (11–14). Such studies are predominantly quantitative in nature, concentrating on objective indicators such as student usage frequency and satisfaction, while there is a lack of research on the subjective attitudes, user experiences, practical challenges, and needs of undergraduate nursing students. Therefore, this study will employ qualitative research methods to thoroughly examine the actual attitudes, user experiences, and practical needs of undergraduate nursing students regarding the use of generative artificial intelligence in their professional studies within the context of Chinese culture. The aim is to provide a foundation for optimizing the development of teaching support systems in nursing programs, cultivating interdisciplinary nursing professionals, and promoting high-quality development in nursing education.

## 2 Methods

### 2.1 Study design and participants

This study will employ a phenomenological qualitative research design and use one-on-one semi-structured in-depth interviews to explore in depth the attitudes and needs of undergraduate nursing students regarding the use of generative artificial intelligence in their professional coursework. This study employed purposive sampling and recruited undergraduate nursing students from three universities in China as participants between January and March 2026. To ensure the accuracy of the data, the design, implementation, and reporting of this study strictly adhered to the Consolidated Criteria for Reporting Qualitative Research (COREQ) (15).

#### 2.1.1 Inclusion and exclusion criteria

The inclusion criteria are: (1) current full-time undergraduate nursing students; (2) individuals who understand the concept of generative AI and have experience using it in their academic studies; (3) individuals who can clearly articulate their personal experiences and views in Chinese and who voluntarily agree to participate in this study. The exclusion criteria are as follows: (1) Individuals with no experience using generative artificial intelligence; (2) Part-time students and those in non-standard enrollment statuses, such as those on leave of absence or extended leave; (3) Newly enrolled students who have not yet begun their core major courses; (4) Individuals with severe communication or cognitive impairments, or those unable to complete the interview due to psychological or physical factors; (5) Individuals who refuse to be recorded or withdraw from the interview midway.

#### 2.1.2 Determination of sample size

The sample size was determined based on the principle of data saturation, meaning that no new themes emerged during the subsequent interviews (16). A total of 24 undergraduate nursing students were invited to participate in this study. Due to scheduling conflicts with their academic schedules, 20 of the invited students agreed to participate and underwent qualitative interviews. Two of these students participated in preliminary interviews; however, since the purpose of the preliminary interviews was to test the scientific validity, rationality, and feasibility of the interview outline—rather than to collect formal data—the content of these interviews was not included in the final research results. By the time the interview reached the 16th student, the topics began to repeat themselves, and no new themes emerged in the subsequent interviews with the 17th and 18th participants. Ultimately, 18 students were included in the study.

### 2.2 Data collection method

First, after obtaining approval from the relevant departments of the university and the college, the researchers posted recruitment posters in the nursing school of the medical college and disseminated recruitment information through academic advisors or class chat groups. The recruitment information briefly outlined the research objectives, interview content, eligibility criteria, estimated interview duration, recording arrangements, confidentiality measures, and the research team's contact information. Students interested in participating should contact the researcher via email. The researcher will evaluate applicants strictly according to the inclusion and exclusion criteria, and those who pass the review will be added to the list of potential participants. Subsequently, research assistants will contact each potential participant individually via email to further explain the research objectives and significance, obtain their consent, have them sign the informed consent form, send them the participant information sheet, and coordinate the interview time and location. Prior to the formal interviews, interviewers will undergo standardized training. Finally, the interviews will be conducted in a quiet conference room or an empty classroom. The interviews will take place in person. The researcher will first collect general demographic information from the participants, including gender, age, grade level, commonly used generative AI tools, frequency of use, and primary use cases. During the interviews, the interviewer strictly followed the interview guide while flexibly employing techniques such as clarification, probing, paraphrasing, summarizing, pausing, and contextual recall based on the specific content of the respondent's answers to guide them in providing detailed accounts of their personal experiences. All interviews will be conducted in Chinese. Each interview will last between 20 and 34 min. With the respondent's consent, the entire interview will be recorded using a digital recording device. Concurrently, a research team member will take field notes to document the interview environment, emotional shifts, pauses, repeated points, nonverbal cues, and preliminary analytical insights. Before the conclusion of each interview, the interviewer will briefly summarize key points and ask the participant to confirm the accuracy of the researcher's understanding to minimize immediate misunderstandings. Interview recordings will be transcribed into verbatim transcripts within 24 h of the interview's conclusion. Upon completion of the transcription, another research team member will cross-check the transcript sentence by sentence against the original recording to correct errors resulting from automatic transcription or manual transcription.

### 2.3 Interview guide

Based on the research topic and objectives, and after reviewing relevant literature, the researchers developed a semi-structured interview guide. Five experts in the relevant field were invited to review it. The expert review focused on the following aspects: consistency between the interview questions and the research objectives; whether the questions were clearly and easily understood; whether the order of the questions followed the respondents' cognitive logic; whether there were any double-barreled or leading questions; whether the questions were overly abstract or sensitive; and whether the overall interview burden was appropriate. The research team compiled the experts' feedback, adopting similar suggestions, while differing opinions were resolved through collective discussion within the research group. Finally, to test the clarity and answerability of the interview guide, as well as the feasibility of the interview process, we conducted pilot interviews with two recruited participants and revised the interview guide based on their feedback. The final version of the interview guide is presented in Table 1.

**Table 1:** Interview guide.

Theme	Question
Understanding generative AI	1. How familiar are you with generative AI? How does it differ from ordinary search engines and educational software?
	2. During your studies, in which stages or tasks do you typically use generative AI?
Current usage and attitudes	3. Have you ever used generative AI tools in your nursing coursework? If so, for how long and how often?
	4. Based on your actual experience using it, what specific challenges in your nursing coursework has it helped you address? How has it impacted your efficiency in studying, your mastery of the material, or the development of your clinical reasoning skills?
	5. What challenges have you encountered, or what concerns or reservations do you have, when using AI-assisted learning?
	6. What is your opinion on the use of generative AI for learning nursing coursework? What are its obvious advantages or disadvantages?
Demand for generative AI	7. In your opinion, what unmet needs do current AI tools have when it comes to answering nursing-related questions or assisting with skill development?
	8. What kind of learning challenges would you like generative AI to help you solve?
Other	9. Do you have any other thoughts or suggestions to add?

### 2.4 Data analysis method

The transcribed text was imported into NVivo 15.0 software for analysis. Data analysis followed Colaizzi's seven-step method. The specific steps included familiarizing oneself with the text, identifying meaningful statements, constructing codes, clustering themes, providing detailed descriptions, generating a basic structure, and validating the structure. The researchers read the data multiple times to familiarize themselves with it. To enhance the credibility of the entire process, two trained researchers conducted the coding independently. When discrepancies arose during the coding process, all researchers resolved them through repeated discussions until a consensus was reached. Finally, to verify whether the identified themes accurately reflected the respondents' genuine views, we contacted 10 respondents for member checking and asked them if the themes accurately reflected their own experiences. None of the participants raised any objections. Furthermore, the entire research team discussed the entire analysis process to ensure that the final themes clearly reflected the interview data.

### 2.5 Researcher reflexivity

The research team consists of nursing educators, clinical nurses, and academic researchers from three regions in China, all of whom possess extensive experience in qualitative research. Prior to formal data collection, the team conducted a standardized reflective workshop to identify and document each researcher's preconceptions regarding generative artificial intelligence. Some team members generally embraced generative AI, viewing it as an effective tool for self-directed learning; others, however, adopted a cautious stance, expressing concerns that it might weaken learners' critical thinking skills and foster academic dependency. To prevent these assumptions from unduly influencing data collection and analysis, the research team maintained reflective journals throughout the interview phase and held regular debriefing sessions to ensure the rigor of the research findings.

### 2.6 Ethical considerations

This study was approved by the Ethics Committee of Yancheng Third People's Hospital (2025-128). In this study, we informed all students of the study's objectives, the content of the interviews, data usage, and privacy protection, and the students voluntarily signed informed consent forms. We also informed them that they could withdraw from the study at any time without providing a reason. Students who did not voluntarily participate or did not consent to being recorded were excluded from the study.

## 3 Result

### 3.1 Demographic characteristics of the participants

A total of 18 eligible undergraduate nursing students participated in this study, including 5 males and 13 females. There were 4 first-year students, 5 second-year students, 6 third-year students, and 3 fourth-year students. The average age of the respondents was 20.22 ± 1.70 years. Commonly used generative AI tools included Doubao (94.44%), Deep Seek (88.89%), Kimi (77.78%), Quark (88.89%), and ChatGPT (11.11%). For further details, see Table 2.

**Table 2:** General information of respondents.

ID	Gender	Age	Grade	Generative AI tools I have used	Frequency of use	Use cases
1	Female	22	Junior	①②③④	3~5 times per week	①②③④
2	Female	24	Senior	①②③④⑤	>5 times per week	①②③④⑤
3	Male	20	Junior	①②④	3~5 times per week	①②③④
4	Female	19	Sophomore	①②③④	3~5 times per week	①②③
5	Female	21	Sophomore	①②③④	>5 times per week	①②③④
6	Female	19	Sophomore	①②③④	< 3 times per week	②③
7	Female	20	Junior	①②③④⑤	>5 times per week	①②③④
8	Male	21	Junior	①②③④	>5 times per week	①②③④
9	Male	21	Senior	①②③④	>5 times per week	①②④⑤
10	Female	23	Senior	①②③④	>5 times per week	①②③④⑤
11	Female	21	Junior	①②③④	3~5 times per week	①②③④
12	Female	21	Junior	①②③④	3~5 times per week	①②③④
13	Female	18	Freshman	①②④	3~5 times per week	①②
14	Female	18	Freshman	①②④	< 3 times per week	①②③
15	Female	20	Sophomore	①②③④	3~5 times per week	①②③④
16	Female	19	Sophomore	①②③④	3-−5 times per week	①②③
17	Male	19	Freshman	①②③	< 3 times per week	①③
18	Male	18	Freshman	②	< 3 times per week	②③

Generative AI tools I have used: Doubao; Deep Seek; Kimi; Quark; and ChatGPT.

Use Cases: Problem-solving; Research; Course support; Academic writing; and Clinical internships.

### 3.2 Theme 1: undergraduate nursing students have a rational attitude toward generative AI

#### 3.2.1 Generative AI can broaden learning perspectives and improve learning efficiency

Generative AI not only improves learning efficiency but also helps broaden students' perspectives to some extent. The field of nursing is characterized by a vast amount of knowledge, complex course content, and heavy academic workload. Students typically need to complete various tasks within a limited timeframe, such as organizing key points from textbooks, analyzing complex case studies, solving homework problems, and completing group assignments. Undergraduate nursing students generally believe that generative AI can quickly handle processes such as knowledge distillation, key point summarization, problem explanation, and information retrieval, thereby reducing the time spent on organizing key points and searching for and filtering materials. Furthermore, when faced with a large volume of textbook information and scattered key points, generative AI helps them quickly get into a learning mindset, clarify the structure of knowledge, and improve learning efficiency. At the same time, some students noted that generative AI can generate a variety of ideas and prompts, helping them overcome creative blocks, prompting them to explore new ways of thinking, and broadening their learning perspectives.

*S3: What might otherwise have been scattered bits of information can now be consolidated by AI, saving us time on organizing it*.

*S8: The main advantage is saving time and improving efficiency. With such a thick book, if you ask the AI to summarize the knowledge structure of a specific section, it can do it in just a few seconds*.

*S13: Generative AI helps me come up with new ideas for my homework. Whenever I'm stuck, it gives me a fresh perspective and inspires me to come up with creative solutions I hadn't thought of before*.

These positive attitudes reflect that Undergraduate nursing students recognize the value of generative AI as a supplement to their professional studies and view it as a tool for broadening their perspectives and enhancing learning efficiency.

#### 3.2.2 Generative AI can help alleviate anxiety

Some undergraduate nursing students have noted that generative AI can help alleviate anxiety to some extent. When faced with questions from instructors or unfamiliar concepts, students often feel nervous due to insufficient preparation, fear of giving the wrong answer, or concern about expressing themselves clearly. Generative AI can help students quickly generate answers and provide ideas, thereby reducing their sense of panic when faced with questions.

*S17: When the teacher suddenly asks a question and I'm not entirely familiar with the topic, I tend to get a little nervous. But after using AI to generate an answer for me, I feel more confident*.

*S14: My foundation isn't very solid, so when the teacher calls on me in class and I encounter a concept I haven't fully grasped, I get really nervous and worry that I won't be able to explain it clearly. But after using AI to organize my answers and thought process in advance, I feel much more at ease, and my anxiety is significantly reduced*.

*S10: When I encounter unfamiliar material, I worry that I'll give the wrong answer and be laughed at by my classmates, but AI helps me organize my thoughts first, so I feel more confident when speaking up in class*.

These findings suggest that generative AI not only provides students with academic support but also serves as an emotional buffer to some extent, thereby increasing students' willingness to use it in their major coursework.

#### 3.2.3 Undergraduate nursing students have concerns about the use of generative AI in their academic studies

Undergraduate nursing students have a range of concerns regarding the use of generative AI in their field. These concerns primarily center on information accuracy, reliance on AI, and academic integrity. In this study, the generative AI tools most frequently used by students included Doubao, DeepSeek, Quark, and Kimi, among others. These tools all exhibit varying degrees of limitations when answering nursing-related questions. Interviews revealed that when using Doubao to answer nursing questions, some students occasionally encountered conclusions that contradicted textbook content or inaccurate numerical values. If students fail to critically evaluate this information, such errors could mislead their understanding of the subject matter. At the same time, because generative AI offers a convenient and quick way to answer questions, some students worry that prolonged use may lead to dependency, thereby diminishing their ability to think independently when addressing problems. Furthermore, the course “Nursing Research” involves learning tasks such as literature searches and writing reviews. Some students pointed out that when using DeepSeek to search for literature, fake documents may appear; if not verified, this could raise issues regarding academic integrity.

*S1: I think the biggest drawback is that Doubao sometimes gives the wrong answers—answers that are completely different from what's in the textbook, and sometimes even the opposite*.

*S7: When I encounter a problem, I sometimes instinctively turn to generative AI for help, and I worry that this habit might undermine my ability to think independently*.

*S11: When using AI to polish this content, if others ask the same question, the answers it provides may be largely consistent, which increases the AI's duplication rate*.

These concerns reflect that undergraduate nursing students have a clear understanding of the potential risks of generative AI in their professional studies.

### 3.3 Theme 2: nursing undergraduates' needs regarding the use of generative artificial intelligence in their academic studies

#### 3.3.1 The need for accurate information

The need for accurate information is one of the most prominent requirements that undergraduate nursing students have for generative AI. Students noted that generative AI is used in a variety of contexts, including researching materials, answering questions, and writing academic papers. In these contexts, the accuracy of information directly impacts the reliability of professional judgment. Therefore, undergraduate nursing students generally expect generative AI to provide more accurate, reliable, and verifiable information to support their professional studies.

*S9: I hope the authenticity of the references it finds can be improved further; it shouldn't fabricate fake references, and I hope they can be found through an online search*.

*S1: I'd like to import all the content from the learning materials into the AI so that it can provide more accurate results when searching for information*.

These findings indicate that nursing undergraduates' expectations of generative AI extend beyond merely accessing information quickly; they place greater emphasis on its professional credibility and the reliability of the knowledge it provides. Rigor is a fundamental requirement in the field of nursing, and generative AI should further enhance the accuracy of the information it generates through technical optimization, thereby providing students with more reliable learning support.

#### 3.3.2 The need for humanistic care

Nursing undergraduates hope that generative AI will provide more personalized, contextual, and individualized support in their studies of psychological nursing and nurse-patient communication. Students believe that current generative AI often produces stiff language and rigid response patterns when dealing with psychological nursing, humanistic care, and communication scenarios, and struggles to adapt its expressions to different patients' emotional states. This makes it difficult for such AI to replicate the emotional responses and nuanced interactions found in real-life communication during simulated nurse-patient interactions, hindering students' ability to gain clinical-like interactive experiences and meet their learning needs for developing humanistic care skills. Therefore, students hope that future generative AI will not only optimize language expression but also provide more natural, empathetic, and practice-oriented support during simulated patient interactions and scenario-based exercises.

*S2: I find it difficult to use generative AI to grasp the emotional responses and communication strategies appropriate for nursing interactions, which affects my understanding of the practice of humanistic care*.

*S15: I hope that in the realm of psychological nursing, it will do more than just make the wording sound smoother; I hope it can provide more appropriate responses based on the patient's emotional state, as this would better approximate real-life communication*.

These findings suggest that undergraduate nursing students hope generative AI will better align with the communication contexts and emotional support needs of nursing practice, thereby aiding in the development of their patient-centered care skills.

#### 3.3.3 The need for external support

The interviews revealed that nursing undergraduates' need for generative AI also extends to external support. This primarily manifests in areas such as curriculum support from the university, faculty guidance, and institutional guidelines. Some students noted that although they frequently use generative AI in their professional studies, they still lack systematic guidance on how to formulate effective queries and apply the technology more appropriately to their nursing studies. At the same time, some soon-to-graduate students expressed a strong desire to master techniques for efficiently using generative AI to assist with the writing of their thesis. Consequently, they hope the university will provide support to help students use generative AI correctly and efficiently. Furthermore, the respondents' expectations for external support extend beyond the mere offering of courses; they hope the university will integrate generative AI into nursing education by aligning it with the practical realities of the field. Students hope the university will provide relevant training and gradually incorporate generative AI into simulation training and clinical scenario-based teaching to better foster the development of professional nursing competencies.

*S3: I hope the school will offer courses that teach us how to better master these AI technologies and how to apply them more effectively to our nursing studies*.

*S12: For example, the school could offer some AI courses to teach us how to use it efficiently, as well as the limitations of AI use and any legal requirements—it would be helpful to learn about those too*.

*S15: While writing my thesis, I realized I was lacking in both technical skills and subject knowledge. I hope there will be skills training courses to help us gain a deeper understanding of AI tools*.

These findings indicate that undergraduate nursing students hope their institutions will provide greater support and assistance in terms of curriculum, guidance, and resources, so that generative AI can be better integrated into nursing education and contribute to the development of students' professional competencies.

## 4 Discussion

This study found that undergraduate nursing students widely use generative AI for answering questions, researching information, academic writing, coursework support, and clinical placements. At the same time, the study indicates that the surveyed students use domestically developed generative AI more frequently. This may be because domestic generative AI offers advantages in terms of localization and ease of use, while accessing large foreign language models often involves certain technical barriers and access thresholds. Through semi-structured interviews, this study revealed nursing students' attitudes toward and needs regarding generative AI in their professional studies. Overall, students expressed approval of generative AI, but they also raised concerns about its use and articulated corresponding expectations.

### 4.1 Generative AI can support nursing undergraduates in their studies and help alleviate psychological anxiety

This study found that undergraduate nursing students demonstrated a high level of acceptance toward generative AI, which effectively addresses both their need for academic support in professional knowledge and their need for anxiety relief on a psychological level. Nursing knowledge encompasses basic medicine, specialized nursing, and complex clinical care plans, characterized by high information density and complex concepts. In this study, students clearly recognized the positive role of generative AI in supporting their learning. Generative AI can quickly summarize reading materials, answer complex questions, propose innovative ideas, construct thesis frameworks, and polish texts, effectively improving students' learning efficiency while providing them with new cognitive pathways. Research has found (17)that students often experience classroom anxiety when answering questions in class due to fear of making mistakes, insufficient preparation, or unfamiliarity with the material. In the classroom, students can use generative AI to quickly review relevant knowledge, organize their thoughts, and refine their reasoning before speaking up, thereby reducing the anxiety caused by inadequate preparation or uncertainty. Similar to our findings, Cai et al. (18) found that introducing generative AI into educational settings can create a safe pre-questioning environment, significantly reducing students' psychological anxiety when faced with questions. However, studies by Jiang et al. (19) and Kang et al. (13) involving nursing graduate students and researchers both found that some respondents experienced “technological displacement anxiety”—that is, concerns that generative AI might ultimately undermine or even replace their professional roles. This finding stands in stark contrast to the results of this study, in which students believed that generative AI helps alleviate classroom-related anxiety. This discrepancy may reflect the fact that undergraduates are still in the early stages of their professional identity development and primarily view generative AI as a practical learning aid, whereas more experienced nursing professionals evaluate it through the lenses of career sustainability, professional value, and research integrity. However, it is worth noting that while generative AI allows for quick access to answers and temporarily alleviates anxiety about being called on in class, if students rely on this coping strategy over the long term, it may mask their actual knowledge gaps. This aligns with the findings of Izquierdo-Condoy et al. (20). Furthermore, overreliance on generated answers to address external evaluations can easily lead students to misjudge their actual professional competence and undermine their ability to make independent decisions (21). Therefore, higher education nursing institutions should further optimize classroom teaching and interaction methods to reduce students' use of generative AI when asked questions, guiding them to apply generative AI to clinical nursing reasoning to ensure that technology truly serves the development of core nursing competencies.

### 4.2 Nursing undergraduates have a clear understanding of the limitations of generative AI

This study found that undergraduate nursing students not only express approval of certain aspects of generative AI but also have a clear understanding of its shortcomings, which primarily include accuracy, dependency, and academic integrity. While generative AI can quickly synthesize information and generate coherent, highly readable responses, its linguistic fluency does not necessarily imply an equivalent level of professional accuracy in the content (22, 23). Nursing education focuses not only on whether information is expressed clearly, and content is adequately synthesized, but more importantly on the ability to form reliable judgments based on textbooks and guidelines. Therefore, students' concerns regarding the lack of accuracy in generative AI information and inconsistencies between the generated content and textbooks reflect not only their worries about the reliability of the technology's output but may also be related to the disciplinary characteristics of the nursing field, which emphasizes intellectual rigor and adherence to standards and norms. Previous research has similarly noted (24)that students' concerns regarding generative AI primarily stem from issues such as insufficient output reliability, opaque sources, and difficulties in fact-checking. Therefore, it is recommended that the application of generative AI in educational settings incorporate human supervision and fact-checking. t the same time, the development of nursing competencies does not depend on the answers themselves, but rather on the prior processes of analysis and judgment. When students analyze clinical cases, develop nursing plans, or write academic papers, they must engage in analysis, comparison, judgment, and reasoning, thereby gradually deepening their understanding of professional knowledge. In this process, relying on generative AI to directly generate content can stifle the thought process (3); not only does this make students prone to dependency, but it also shifts their thinking from active to passive, thereby undermining their critical thinking, problem-solving skills, and ability to make independent judgments. Previous research has confirmed this view (25), suggesting that overreliance on generative AI may weaken students' critical thinking skills and reduce their motivation to learn. Therefore, in educational practice, greater emphasis should be placed on guiding students to use generative AI appropriately, treating it as a tool to aid their thinking and better support their knowledge construction. Furthermore, this study found that students expressed concerns about fabricated literature and the homogenization of articles when using generative AI to write papers. These concerns may extend beyond academic misconduct to encompass issues of evidentiary responsibility and authorial accountability in the writing process. If students rely heavily on AI-generated content during the processes of literature review, data synthesis, and text composition, their substantive engagement in verifying sources, constructing arguments, and expressing academic ideas will diminish—even if the resulting text appears more complete in form and content (1)—leading to a decline in their academic writing skills.

### 4.3 Addressing the needs of undergraduate nursing students regarding generative AI to better integrate it into nursing education

Undergraduate nursing students have several unmet needs regarding the integration of generative AI into nursing education, primarily in terms of information accuracy, humanistic care, and external support. Students' needs for generative AI in educational settings are mainly focused on content reliability, support for contextual communication, and teaching assistance. In this study, nursing undergraduates expressed a particularly strong need for information accuracy. This may be related to the high standards for knowledge authenticity and professionalism in nursing education, as well as the lack of authoritative information support when students use generative AI. This finding is consistent with the results of Han et al. (26). At the same time, students have expressed a strong need for generative AI to support humanistic care. They hope that generative AI will provide responses that are more closely aligned with nursing practice in patient-nurse communication, psychological support, and situational interactions, rather than merely outputting standardized information. However, research indicates (8) that current generative AI still has shortcomings in humanistic communication, primarily manifested in limited emotional understanding, formulaic responses, and insufficient sensitivity to complex nursing situations. The findings of this study further highlight these shortcomings. Regarding emotional understanding, students noted that generative AI struggles to accurately grasp emotional shifts in nursing communication and the corresponding communication strategies. It still has certain limitations in emotional perception and contextualized responses, making it difficult to promptly adjust responses based on changes in the patient's emotional state. Regarding emotional simulation, students believe that interactions with generative AI tend to be more one-way information output rather than dynamic, two-way emotional exchanges, and thus have certain limitations in helping students practice sustained emotional perception and communication adjustments. Therefore, it is recommended that generative AI be incorporated into nurse-patient communication training and scenario simulations, with faculty guidance used to enhance its applicability in teaching humanistic care. At the same time, the auxiliary role of generative AI should be emphasized to prevent its generated discourse from replacing genuine emotional responses and interpersonal interactions in nursing practice. Additionally, students have expressed a need for external support. They hope to receive course guidance, faculty guidance, and institutional support during the use of generative AI. However, existing research indicates (27) that a lack of support is prevalent in current nursing education, primarily manifested in students' unclear understanding of usage boundaries, insufficient faculty guidance, and a lack of systematic training. Students participating in this study also reported that, when using the generative AI tools commonly available in China today, some are unsure how to ask appropriate questions to obtain more accurate professional information, and lack the ability to assess the authenticity of the generated content. This suggests that more targeted guidance and training should be provided, tailored to the specific domestic generative AI tools students actually use, to help them improve their information literacy and awareness of proper usage. Therefore, it is recommended to establish a continuous support system for integrating generative AI into nursing education. By leveraging digital platforms to integrate curriculum resources, case studies, and standardized training content, efforts should be made to strengthen faculty training, curriculum integration, and resource sharing to facilitate the better integration of generative AI into nursing education.

## 5 Limitations

This study has several limitations. First, this study employed purposive sampling, with respondents drawn exclusively from three institutions in China, resulting in a relatively small sample size. Given the differences among universities in various regions regarding teaching resources, levels of digitalization, and policy orientations toward generative AI, the generalizability of the findings to other regions or institutions may be limited. Second, as this is a qualitative study, data collection and analysis may have been influenced by subjective factors. Third, the data primarily comes from semi-structured interviews and relies on self-reports from respondents, meaning their answers may be influenced by subjective factors. Fourth, the vast majority of participants in this study have only used domestic generative AI tools, while only a small number have experience with foreign generative AI tools. Domestic tools may differ from their Western counterparts in terms of functional design, training data, and content mechanisms, which to some extent limits the generalizability of the study's findings to other tool ecosystems or cultural contexts. Finally, this study focuses solely on undergraduate nursing students with a small sample size and does not include faculty members or clinical instructors, which may result in incomplete findings. Therefore, future research should be conducted in different regions with a larger sample size to explore the application of generative AI in nursing education from the perspectives of faculty members and clinical instructors.

## 6 Conclusion

This study found that undergraduate nursing students generally adopt a rational and accepting attitude toward generative AI in their professional studies. While recognizing its value as a learning aid, they maintain a clear understanding of issues related to information accuracy, overreliance, and academic integrity. In terms of needs, students expressed a strong desire for information accuracy, humanistic care, and external support. Therefore, it is recommended that nursing institutions provide support to better integrate generative AI into nursing education and guide students in developing sound habits regarding AI use, thereby promoting the high-quality training of nursing professionals.

## Data Availability

The original contributions presented in the study are included in the article/supplementary material, further inquiries can be directed to the corresponding authors.
